# Inhibition, flexibility, working memory and planning in autism spectrum disorders with and without comorbid ADHD-symptoms

**DOI:** 10.1186/1753-2000-2-4

**Published:** 2008-01-31

**Authors:** Judith Sinzig, Dagmar Morsch, Nicole Bruning, Martin H Schmidt, Gerd Lehmkuhl

**Affiliations:** 1 Department of Child & Adolescent Psychiatry and Psychotherapy, University of Cologne, Robert-Koch-Strasse 10, D-50931 Cologne, Germany; 2 Department of Child & Adolescent Psychiatry and Psychotherapy, Central Institute of Mental Health, P.O. Box 122120, 68072, Mannheim, Germany

## Abstract

**Background:**

Recent studies have not paid a great deal of attention to comorbid attention-deficit/hyperactivity disorder (ADHD) symptoms in autistic children even though it is well known that almost half of children with autism spectrum disorder (ASD) suffer from hyperactivity, inattention and impulsivity. The goal of this study was to evaluate and compare executive functioning (EF) profiles in children with ADHD and in children with ASD with and without comorbid ADHD.

**Methods:**

Children aged 6 to 18 years old with ADHD (n = 20) or ASD (High-Functioning autism or Asperger syndrome) with (n = 20) and without (n = 20) comorbid ADHD and a typically developing group (n = 20) were compared on a battery of EF tasks comprising inhibition, flexibility, working memory and planning tasks. A MANOVA, effect sizes as well as correlations between ADHD-symptomatology and EF performance were calculated. Age- and IQ-corrected z scores were used.

**Results:**

There was a significant effect for the factor group (F = 1.55; dF = 42; p = .02). Post-hoc analysis revealed significant differences between the ADHD and the TD group on the inhibition task for false alarms (p = .01) and between the ADHD group, the ASD+ group (p = .03), the ASD- group (p = .02) and the TD group (p = .01) for omissions. Effect sizes showed clear deficits of ADHD children in inhibition and working memory tasks. Participants with ASD were impaired in planning and flexibility abilities. The ASD+ group showed compared to the ASD- group more problems in inhibitory performance but not in the working memory task.

**Conclusion:**

Our findings replicate previous results reporting impairment of ADHD children in inhibition and working memory tasks and of ASD children in planning and flexibility abilities. The ASD + group showed similarities to the ADHD group with regard to inhibitory but not to working memory deficits. Nevertheless the heterogeneity of these and previous results shows that EF assessment is not useful for differential diagnosis between ADHD and ASD. It might be useful for evaluating strengths and weaknesses in individual children.

## Background

Autism spectrum disorders (ASD) and attention-deficit/hyperactivity disorder (ADHD) are childhood-onset neurodevelopmental disorders affecting key fronto-striatal and fronto-parietal circuits that are important for executive functions [1,2]. The term executive function (EF) is used in brain research and neuropsychology to describe mental functions with which higher life forms govern their behaviour. EFs involve multiple distributed neural networks that include the thalamus, basal ganglia and prefrontal cortex [3,4].

Several authors have proposed that symptoms of ADHD arise from a primary deficit in a specific EF domain such as response inhibition, working memory, or a more general weakness in executive control [5,6]. This hypothesis is based on the observation that prefrontal lesions sometimes produce behavioural hyperactivity, distractibility or impulsivity as well as deficits on EF tasks [7]. A theory by Barkley considered inhibitory dysfunction as a core deficit in children with ADHD, which causes secondary deficiencies in other EFs such as working memory, cognitive flexibility and planning [8]. Nigg describes in a meta-analysis of neuropsychological findings in ADHD highest effect sizes for spatial working memory and response suppression tasks (ADHD vs. Non-ADHD children) [9].

There are also many empirical reports of executive impairments in individuals with autism spectrum disorders across wide age ranges and functioning levels [10,11]. Hill's recent review highlights impairments on at least two aspects of EF: planning and flexibility [2].

EFs have been examined in neuropsychological studies that were carried out in direct comparison of children and adolescents with ASD or ADHD. To date six studies have compared EF in ASD and ADHD.

Two studies were conducted independently in the year 1999 by Ozonoff et al. and Nyden et al. [12,13]. Ozonoff et al. found in children with ASD difficulties in planning and cognitive flexibility but no inhibition deficit, and the reverse neuropsychological pattern in children with ADHD. Nyden et al. were not able to replicate these findings. In their study, both groups of disorders showed an inhibition deficit, and the ADHD children had a limited cognitive flexibility.

Geurts et al. extended the aforementioned studies by examining a broader spectrum of EFs in patients with ADHD and high-functioning autism (HFA) with the aim of distinguishing between the two disorders [14]. The ASD-group showed deficits on all EF tasks except of interference control and working memory, and significantly greater impairment than the ADHD-group on planning and cognitive flexibility. The ADHD group was most impaired on inhibition of prepotent response and verbal fluency.

Goldberg et al. report no differences between ADHD and ASD children on response inhibition, planning and flexibility tasks [15]. Both groups were impaired on a working memory task compared to healthy control children.

Happé et al. compared age- and IQ-matched groups with ASD and ADHD and found greater inhibitory problems in the ADHD group on a Go/NoGo, planning and working memory task, while the ASD group was solely worse on a response selection task [16].

A study by Johnson et al. tested children with HFA and ADHD on a Sustained Attention to Response Task (SART) and report of clear deficits in response inhibition and sustained attention in the ADHD group. The HFA group showed dissociation in response inhibition performance [17].

The results of the studies differed partly. A reason for that might be the differences in the age ranges within the sample and the different types of tasks that were applied, whereas mean age and IQ were similar. Table 1 summarizes assessment procedures and sample characteristics of these previous studies.

**Table 1:** Previous studies comparing executive functions in ASD vs. ADHD

	**Ozonoff et al. 1999**	**Nyden et al. 1999**	**Geurts et al. 2004**	**Goldberg et al. 2005**	**Happé et al. 2006**	**Johnson et al. 2007**
**Sample (n)**	n = 93	n = 30	n = 136	n = 70	n = 94	n = 62
-Autism	40	10	41	17	32	21
-ADHD	24	10	54	21	30	23
-TD	29	10	41	32	32	18
**ASD diagnosis (%)**						
- HFA	40 (100)	-	41 (100)	17 (100)	6 (19)	21 (100)
- AS	-	10 (100)	-	-	26 (81)	-
**ADHD subtype (%)**	?	?		?	?	
- Combined s.			36 (67)			22
- Inattentive only s.			16 (29)			1
- Hyperactive/Impulsive s.			2 (4)			-
**Inclusion of ADHD in ASD group**	?	?	Yes,	no	no	no
			only inattentive subtype			
**Age at testing (years)**	12.1	10.0	9.3	11.2	10.1	11.2
**Min-Max**	(6–18)	(8–11)	(6–13)	(?)	(8–12)	(?)
**IQ**	103.4	95.6	103.1	107.6	101.8	101.2
**Neuropsychological measures**						
-Inhibition	Stroop CWT	Go-No-Go, RIT	CT, CDT, TEA-Ch	Stroop CWT	Go-No-Go	SART
-Working Memory	-	-	S-OPT	C SWM	C SWM	-
-Planning	TOH	-	TOL	C SOC	C SOC	-
-Flexibility	WCST	WCST	WCST	C ID/ED	C ID/ED,	-
					Verbal Fluency	

Note: CDT = Circle Drawing Task; C ID/ED = CANTAB Intra-dimensional/extra-dimensional shift task; C SOC = CANTAB Stockings of Cambridge; C SWM = CANTAB Spatial WorkingMemory; CT = Change Task; RIT = Response Inhibition Task; SART = Sustained Attention to Response Task; S-OPT = Self-Ordered Pointing; Stroop CWT = Stroop Colour Word Test; TD = Typically developing group; TEA-Ch = Test of Everyday Attention for Children; TOH = Tower of Hanoi; TOL = Tower of London; WCST = Wisconsin Card Sorting Test;

ADHD is still an exclusion criterion for Pervasive Developmental Disorders in ICD-10 and DSM-IV-TR even though there is preliminary evidence of genetic linkage in both disorders at chromosomal locations 2q24 and 16p13, 16p1, 17p11 and 5p13 as well as 15q [20-22]. Furthermore neuroimaging studies show anomalies in fronto-striatal and cerebellar structures in both ADHD and ASD [23,24].

To date, studies dealing with the topic of EF deficits in both ASD and ADHD have not devoted a great deal of attention to comorbid ADHD symptoms in the autistic participants, although several authors have described that almost half of the autistic children suffer from comorbid hyperactivity, impulsivity and inattention [18,19]. In the studies by Goldberg et al. and Happé et al. autistic children with ADHD were excluded [15,16]. Geurts et al. only included autistic children with the inattentive ADHD subtype [14]. The sample of Johnson et al. comprised 12 (57%) children with HFA, scoring at least 65 on the Conners' ADHD Index, that were not treated as a subgroup in the statistical analysis [17]. Ozonoff et al. and Nyden et al. don't even mention about the rates of ADHD symptoms in the ASD groups of their studies [12,13].

The present study aimed to assess the impact of comorbid ADHD-symptoms in children with HFA or Asperger syndrome on the ability on EF tasks. For this purpose, we compared autistic children with and without comorbid ADHD symptoms, children with ADHD and normal healthy children on four EF tasks: inhibition, planning, spatial working memory and flexibility. To the authors' knowledge this is compared to previous studies comparing ADHD and ASD samples the first study including both a pure ASD group AND an ASD with comorbid ADHD group.

We predicted that profile differences might exist, with ADHD children being more impaired in inhibition and working memory and children with ASD showing greater difficulties in flexibility and planning referring to the above mentioned studies by Nigg and Hill [2,3]. With regard to the studies using equal test batteries we especially expected errors of omission and commission on the Go/NoGO task as described by Happé et al. and working memory deficits of the parameter "errors" of the CANTAB as described by Goldberg et al. and Happé et al. [15,16].

Additionally it was hypothesized that the ASD group with comorbid ADHD symptoms performs worse by an order equivalent to the addition of each disorder individually (additivity hypothesis) and not worse than the combination of the two disorders (over-additivity hypothesis) or similarly to either of the disorders on its own (under-additivity hypothesis) [25].

## Methods

The total sample of this study consisted of four subgroups. The ASD with comorbid ADHD symptoms group (ASD+) comprised 19 boys and one girl with a diagnosis of either HFA (n = 5) or an Asperger syndrome (n = 15), the ASD without comorbid ADHD symptoms group (ASD-) comprised 16 boys and 4 girls with a high-functioning diagnosis (n = 5) or an Asperger diagnosis (n = 15). The ADHD group consisted of 19 boys and one girl. Also children with a diagnosis of predominantly inattentive type were included. The typically developing (TD) comparison group comprised 14 boys and 6 girls that were recruited through schools, family friends of participants in the clinical groups or personal contacts. Children were not included if they had any psychiatric diagnosis or family history of social or attention related problems.

The participants were required not to be taking any central nervous system active medication except for methylphenidate. All were required to be off medication for at least 24 hours prior to the administration of the experimental tasks. This period is described to be sufficient to ensure full wash-out [26]. More participants in the ADHD group (n = 15; 75.0%) than in the ASD+ group (n = 7; 38.9%) were treated with medication.

Furthermore the participants were required to have an IQ ≥ 80. Comorbid Oppositional Defiant Disorder (ODD) was allowed in both clinical groups. This inclusion was because findings from studies suggest that ADHD associated with conduct disorder (CD) may be a distinct subtype, but this does not appear to be the case for ADHD associated with ODD [27]. Participants with known medical causes of autism, including fragile X syndrome and tuberous sclerosis, and those with other neurological disorders, e.g. epilepsy, were excluded.

Table 2 summarizes the clinical and demographic features of the sample.

**Table 2:** Clinical and Demographic features of the Sample

	**ASD+**	**ASD-**	**ADHD**	**TD**	**Group effect**		**Post hoc Scheffé**
	**(n = 20)**	**(n = 20)**	**(n = 20)**	**(n = 20)**	**F**	**p**	
**No. (%)**							

**Gender**					2.4	0.8	
-male	19 (94.7)	16 (80.0)	19 (95.0)	14 (70.0)			
-female	1 (5.3)	4 (20.0)	1 (5.0)	6 (30.0)			
**Autism diagnosis**					0	1.0	
- Asperger syndrome	15 (75.0)	15 (75.0)	-	-			
- High-functioning autism	5 (25.0)	5 (25.0)					
**ADHD subtype**					2.0	0.2	
- Combined s.	7 (35.0)	-	8 (40.0)	-			
- Inattentive only s.	11 (55.0)		11 (55.0)				
-Hyperactive/Impulsive s.	2 (10.0)		1 (5.0)				
**Comorbid ODD**	9 (45.0)	6 (30.0)	11 (55.0)	-	1.3	0.3	

**Mean (SD)**							

**Age at testing (years)**	10.9 (3.1)	14.3 (3.0)	12.2 (2.0)	13.1 (3.0)	4.4	0.1	ASD+ < ASD-**
	(6.0–17.0)	(8.3–18.9)	(7.1–17.6)	(7.6–17.6)			
**IQ**	103 (13.0)	112 (17.7)	98(13.4)	113 (11.9)	5.7	0.01	ADHD < TD**, ASD-**
**Min-Max**	(80–131)	(82–146)	(80–128)	(95–144)			

*Post hoc Test p < .05; **Post hoc Test p .01; ***Post hoc Test p < .001

Note: ADHD = attention-deficit/hyperactivity disorder; TD = typically developing group; CD = conduct disorder; ODD = oppositional defiant disorder.

### General Procedure

The participants were recruited from our inpatient and outpatient department of child and adolescent psychiatry, while the healthy control group consisted of healthy siblings of the patients or were other children interested in taking part. All new referrals with suspected ADHD or ASD underwent an extensive child psychiatric examination, which was conducted by an experienced child and adolescent psychiatrist according to DSM-IV-TR criteria. Additionally standardized psychopathological measures were used (see diagnostic measures section). IQ was measured using the Culture Fair Intelligence Test, a non-verbal one-dimensional IQ-test [28]. The diagnosis of ADHD in the ASD+ group was given before recruitment. All children from the ASD+ group met full DSM-IV-TR criteria and were excluded if they had subthreshold ADHD characteristics. They furthermore fulfilled the age and the pervasiveness criterion as required in DSM-IV-TR.

Informed parental consent was obtained for all participants, and the study was approved by the Medical Ethical Committee of the University of Cologne. All children were tested individually in the Department of Child & Adolescent Psychiatry in a quiet room by one of the two researchers. The person testing was blind with regard to the ADHD diagnosis of the autistic participants. Testing was carried out within a larger study that comprised a two-hour session. EF tasks were presented in a fixed order (Inhibition, SWM, SOC and ID/ED) approximately after the implementation of the first half of the test. Due to the small simple size we decided not to counterbalance the order of the test. Participants were informed that they could discontinue testing at any time and were given positive comments throughout the testing. The parents or caregivers were sent detailed reports on their child's performance on the tests.

### Diagnostic measures

The diagnosis of autistic disorder was made using the *Autism-Diagnostic Interview-Revised *(*ADI-R*; Cut-offs: Impairment of Social Interaction = 10; Impairment of Communication = 8; Stereotyped Behavior = 3) and the *Autism Diagnostic Observation Scale (ADOS*, Cut-offs: Communication and Social Interaction = 7 (Module 1), 12 (Module 2), 10 (Module 3+4)) [29-32]. Furthermore the *Diagnostic Checklist for Pervasive Developmental disorders (DCL-TES)* was applied, mainly to exclude ASD in the ADHD children and to differentiate between HFA and Asperger syndrome within the ASD group [33]. Additionally the *Diagnostic Checklist for Oppositional Defiant or Conduct disorders (DCL-SSV*) was used to have a dimensional description of ODD-symptoms in both the ASD and the ADHD group.

The diagnosis of attention deficit/hyperactivity disorder was made using the *Diagnostic Checklist for Hyperkinetic Disorders/ADHD (DCL-HKS)*. Similar rating scales have been developed in the United States, based solely on DSM-IV criteria for ADHD [35]. The number of DSM-IV criteria fulfilled was provided, as was the severity score for each item ranging from 0 to 3 [34]. The checklists were applied as an interview with parents and teachers. All three checklists are made up of components of the Diagnostic System for Mental Disorders in Childhood and Adolescence (DISYPS-KJ) based on ICD-10 and DSM-IV and allow the assessment of a dimensional score and a categorical diagnosis [33]. The cut-offs of the checklists correspond with the criteria that have to be fulfilled according to ICD-10 and DSM-IV.

**Figure 1:**
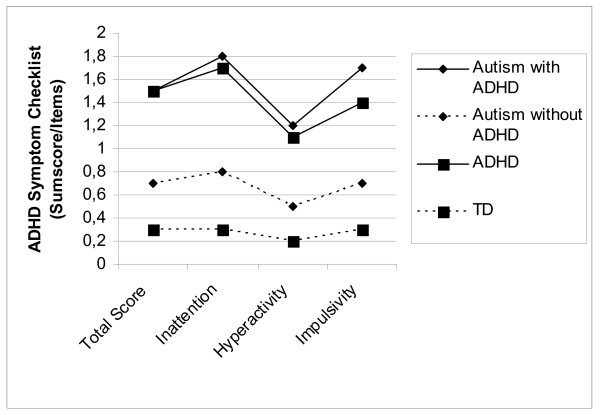
Mean Total scores of ADHD- symptoms (DCL-HKS, DISYPS) for the four diagnostic groups.

**Figure 2:**
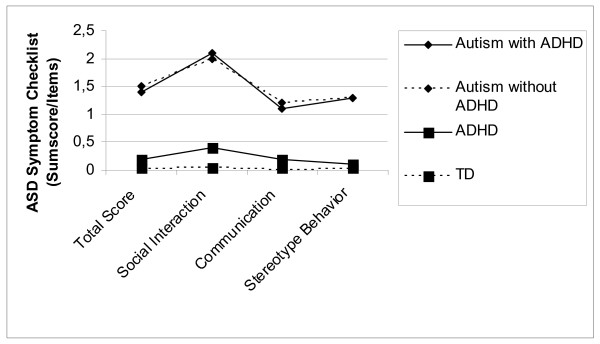
Mean Total scores of ASD-symptoms (DCL-TES, DISYPS) for the four diagnostic groups.

The groups ASD + and ADHD show an equal profile without statistically significant differences in the DCL-HKS scores with regard to the criteria of hyperactivity, inattention and impulsivity. Scores of the DCL-TES were as expected high for both ASD groups and low for the ADHD and the TD group. The scores are illustrated in Figure 1 and 2, separately for the four different groups.

Additional comorbid disorders (emotional disorders, OCD, enuresis and encopresis as well as ODD and OCD) were assessed using the *Kiddie-SADS – Lifetime-Version (K-SADS-PL) *[36]. No relevant comorbid disorders except of ODD, especially no learning disabilities, were found in any of the four groups. The K-SADS was also used to additionally confirm the ADHD diagnosis.

### Experimental Procedure

#### Inhibition

The inhibition (Go/NoGo) task was administered from the "Test for Attentional Performance" (TAP) [37]. In a Go/NoGo condition, two stimuli were presented 40 times in succession (20+ and 20×). The child was asked to press the "yes" key only when a cross (+) appeared. The interstimulus interval was variable from 2150 to 3350 ms and the presentation duration of one stimulus was 200 ms. The dependent measures were the number of misses, false alarms, hits, and the median of RTs of hits. Median was chosen due to the skewness of the RT distributions.

Flexibility, working memory, and planning functions were assessed using sub-tests from the "Cambridge Neuropsychological Automated Test Battery" (CANTAB). This test battery has been employed internationally for 15 years. This battery has already been used by investigators to assess EF in children with normal development, as well as with developmental disorders including autism and ADHD [38-41]. We chose three tasks from the CANTAB, which have already been used in studies assessing children with autism and ADHD: The Stockings of Cambridge Task (SOC), similar to the Tower of Hanoi (TOH), the Spatial Working Memory Task (SWM), and the Intra-Dimensional/Extra-Dimensional Shift Task (ID/ED), similar to the Wisconsin Card Sorting Test (WCST).

#### Intra-Dimensional/Extra-Dimensional Shift (ID/ED)

This task measures the ability to attend to specific attributes of compound stimuli, shifting attention from one attribute to another when required. Participants are presented with a series of multidimensional stimuli, consisting of shapes and lines. In stages 1 through 5 of the task, the discrimination and learning stages, participants learn through trial and error to respond selectively to one specific shape, ignoring the other shape and the lines. In stage 6, the intradimensional shift, new shapes and lines, are introduced, but shape continues to be the salient response dimension. In stage 7 the intradimensional reversal, the previously nonreinforced shape now becomes the correct response. At stage 8, during the critical extradimensional shift, however, the correct rule now changes to the other dimension (e.g., the line) that has been irrelevant for the preceding dozens of trials. Finally in stage 9, the extradimensional reversal, participants must respond to the previously non reinforced line. The dependent measures were the number of errors committed and the number of trials taken to achieve criterion on stages 6 through 9. When participants failed to achieve criterion (six consecutive correct responses) at a given stage, the test was failed and the maximum number of errors (25) was recorded for all subsequent stages not administered.

#### (Spatial-) Working Memory (SWM)

In this test a trial begins with a number of coloured squares being shown on the screen. The overall aim is that the participant should find a blue "counter" in each of the squares and use them to fill up an empty column on the right hand side of the screen. The child must touch each box in turn until one opens with a blue "counter" inside (a search). Returning to an empty box already sampled on this search is an "between-search error". A "Strategy score" is estimated from the number of searches that start from the same location. The dependent measures were the number of between-search errors, strategies and test duration.

#### Planning (Stockings of Cambridge, SOC)

This is a computerized test of spatial planning based upon the "Tower of London" Test. The participant is shown displays containing three coloured balls. The displays can easily be perceived as stacks of coloured balls (one green, one blue and one red) held in stockings or socks suspended from a beam. The participant must use the balls in the lower display to copy the pattern shown in the upper one. The dependent measures were the number of problems solved in the minimum number of moves, mean of the mean initial thinking time, mean of the subsequent thinking time and test duration.

### Statistical Analysis

The statistical analysis was carried out using the SPSS for Windows Program Version 14.0.

In order to examine group differences between the groups a MANOVA with all EF parameters of the four paradigms as the dependent measures and the group as the between-subject variable and additional post hoc Scheffé tests were calculated. Due to the small sample sizes effect sizes, according to Cohen, were calculated in order to examine group differences between the four groups for the EF parameters of the four paradigms [42]. Since a large number of statistical tests were performed, significant results may have capitalised on chance and the overall probability of a type I error likely exceeded 5%. In the case of a priori predictions, Howell argues that correction for multiple comparisons is not warranted [43].

Next, Pearson correlations were carried out between the different executive variables and the values of the DISYPS ADHD scales (ADHD total score, inattention, hyperactivity and impulsivity) and the DISYPS ASD scales (ASD total score, mean impairment of social interaction, mean impairment of communication and mean stereotype behavior) for the four diagnostic groups.

## Results

It might be assumed that neuropsychological performances probably improve due to physiological brain maturation. Also IQ deficits are described as being associated with neuropsychological performance [39,44]. Therefore, we looked for effects of age and IQ before starting the statistical analysis. As a MANOVA with all the dependent measures of the four paradigms and with age and IQ as the between-subject variable revealed significant main effects for age (F = 5.19; p < .00) and IQ (F = 3.08; p = .001) all neuropsychological data were converted using regression analysis with regard to age and IQ value and finally z-transformed (with a mean of 0 and SD of 1) based on the mean and standard deviation of the whole control group [45,46]. By this a comparison of the different variables were additionally facilitated and could be shown on the same scale. The Z scores were calculated so that a positive score reflected good executive performance and vice versa.

Because of these results, we calculated group differences between the four diagnostic groups using a MANOVA with post-hoc Scheffé tests with age and IQ as the dependent variable and group as the between-subject variable. There was a significant group effect for age (F = 4.41; p < .007) as well as for IQ (F = 5.72; p < .01). Post-hoc tests revealed a significant effect for age between the groups ASD+ vs. ASD- (p = .01) and for IQ between the groups ADHD vs. ASD- (p = .01) as well as the TD group (p = .01).

### Executive function tests

Table 3 shows descriptive statistics (mean, standard deviation) and results of a MANOVA and post hoc Scheffé tests with all EF parameters of the four paradigms as the dependent measures and group as the between-subject variable. There was a significant effect for the factor group (F = 1.55; dF = 42; p = .02). Furthermore effect sizes describing the degree of differences of the performance between the four groups on the applied tasks are presented.

**Table 3:** Performance in all attention tasks separated for the four diagnostic groups (mean/SD)

**TASK**	**ASD +**	**ASD -**	**ADHD**	**TD**	**Group effects**		**Effect Sizes**
					**F**	**p**	
**Inhibition (Go/NoGo)**							
Median	0.01 (2.76)	0.39 (1.97)	1.51 (2.24)	0.00 (1.0)	2.26	.09	ADHD < TD***, ADHD < ASD-, ASD+**
Hits	- 1.27 (2.41)	- 0.65 (1.99)	- 1.82 (2.68)	- 0.05 (1.0)	2.56	.06	ADHD < ASD-, TD**; ASD+ < TD**
False alarms	- 0.12 (1.06)	- 0.19 (1.28)	- 1.21 (1.15)	0.01 (1.0)	4.78	.004	ADHD < ASD+, ASD-, TD***
Omissions	- 0.81 (1.41)	- 0.02 (1.28)	- 1.42 (1.61)	0.02 (1.0)	5.02	.003	ADHD < ASD-, TD***; ASD+ < ASD-, TD**
**Flexibility (ID/ED)**							
Stages	0.47 (0.89)	0.31 (0.74)	0.16 (1.01)	- 0.05 (1.0)	1.16	.33	TD < ASD+ **
Errors	0.02 (1.32)	0.23 (1.07)	- 0.06 (1.71)	0.00 (1.0)	0.11	.94	-
Test duration	- 0.83 (1.94)	0.02 (1.21)	- 0.68 (2.36)	0.01 (1.0)	1.28	.28	ASD+ < ASD-, TD**
**Working Memory (SWM)**							
Errors	- 0.32 (1.22)	- 0.62 (1.31)	- 0.88 (0.87)	0.01 (1.0)	2.33	.08	ADHD < TD***, ASD- < TD**
Strategies	0.27 (1.33)	- 0.15 (1.26)	- 0.51 (0.72)	0.01 (1.0)	1.79	.15	ADHD < ASD+, TD**
Test duration	- 0.51 (1.47)	- 0.06 (0.94)	- 0.37 (0.78)	0.01 (1.0)	0.92	.43	-
**Planning (SOC)**							
MITT	0.07 (1.09)	- 0.53 (1.91)	0.39 (1.15)	0.00 (1.0)	1.61	.19	ASD- < ADHD**
MSTT	- 0.53 (4.12)	- 1.01 (2.58)	- 0.33 (3.22)	0.00 (1.0)	0.39	.75	ASD- < TD**
Problems solved	0.46 (1.18)	0.09 (1.31)	0.05 (1.11)	0.01 (1.0)	0.68	.56	-
Test duration	- 0.91 (2.38)	- 0.54 (1.87)	- 0.32 (1.45)	0.07 (1.0)	1.26	.29	ASD+ < TD**

Effect sizes: (mean-differences in independent groups)*d > 0.2; **d > 0.5; ***d > 0.8

Note: ASD+ = ASD with ADHD; ASD- = ASD without ADHD; ADHD = attention-deficit/hyperactivity disorder; TD = Typically developing group, MITT = Mean Initial thinking time; MSTT = Mean subsequent thinking time

#### Inhibition task (Go/NoGo-Task)

On the inhibition task the ADHD group appeared more impaired than and the TD group on all variables with high effect sizes (median: d = 0.9; hits: d = 1.5; false alarms: d = 1.1; omissions: d = 1.2). But also compared to the ASD+ group (median: d = 0.6; false alarms: d = 1.0) and the ASD- group (median: d = 0.5; hits: d = 0.5; false alarms: d = 0.8; omissions: d = 1.0) they performed significantly worse. However the ASD+ group performed less well than the TD (more errors of omission: d = 0.7 and fewer hits: d = 0.7) and the ASD- group (more errors of omission: d = 0.6).

Significant group differences were found for the variable false alarms (F = 4.78; p = .004) and omissions (F = 5.02; p = .003) with post hoc group differences between the ADHD and the TD group for false alarms (p = .01) and between the ADHD group, the ASD+ group (p = .03), the ASD- group (p = .02) and the TD group (p = .01) for omissions.

#### Intra-Dimensional/Extra-Dimensional Shift Task (ID/ED)

The flexibility task was more difficult for participants with ASD and comorbid ADHD symptoms. They made more errors and needed more time for the task compared to the ASD- group (d = 0.6) and the TD group (d = 0.6), but completed more stages compared to the TD group (d = 0.6). The best performance was shown by the ASD- group. Test duration was also longer for the ADHD group with a small effect size (d = 0.4).

No statistically significant differences could be found between any of the groups.

#### Spatial Working Memory Task (SWM)

Participants of the ADHD group performed significantly worse making more errors than the TD group (d = 1.0) as well as needing more strategies than healthy control children (d = 0.7) and autistic children with comorbid ADHD symptoms (d = 0.7). Also the ASD- group made more errors than the TD group (d = 0.6). Furthermore, the ASD+ and the ADHD group needed longer to perform the whole task compared to the ASD- (d = 0.4) and the TD group (d = 0.4). There were no significant group differences on the basis of the MANOVA.

#### Planning Task (Stockings of Cambridge, SOC)

There was a medium effect size between the groups ASD- and ADHD (d = 0.6). All clinical groups needed more time between the subtasks. There was a high effect size between the groups ASD- and the control group (d = 0.6). Participants of the ASD+ group had a longer test duration than those of the TD group (d = 0.6).

There were no significant group differences for any of the tasks.

**Figure 3:**
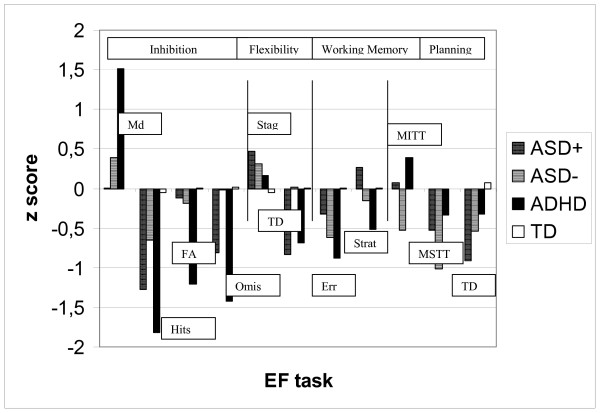
Executive functioning z score plots for significant effect sizes for the four diagnostic groups. Note: Md= Median, FA= False Alarms, Omis= Omissions, Stag= Stages, TD= Test Duration, Err= Errors, Strat= Strategies, MITT= Mean Initial thinking time; MSTT=Mean subsequent thinking time.

The Z score plots with medium and high effect sizes between the four groups are shown in Fig. 3.

### Relationship between EF and ADHD/ASD symptoms

In addition, Pearson product-moment correlations separated for the two groups affected by ADHD-symptoms were used to examine the relationship between all dependent measures of the neuropsychological paradigms and the clinically observed ADHD symptoms (inattention, hyperactivity, impulsivity and ADHD total score) measured with the DCL-HKS.

The ADHD group showed only small, but significant correlations for the variable "flexibility errors" with inattention (r = -0.5, p < .03) and for the variable "flexibility test duration" with inattention (r = -0.5, p < .02). In the ASD+ group, the variable "inhibition median" correlated significantly with the total score ADHD (r = -0.6, p < .01) and impulsivity (r = -0.6, p < .03), the variable "inhibition hits" with inattention (r = -0.6, p < .01), the variable "inhibition false alarms" with hyperactivity (r = -0.5, p < .02) and the total score ADHD (r = -0.5, p < .02) as well as the variable "inhibition omissions" with inattention (r = -0.5, p < .04).

Furthermore the ASD+ group showed small, but significant correlations for the variable "flexibility errors" with inattention (r = -0.6, p < .03) and the variable "working memory test duration" and the total score ADHD (r = 0.5, p < .02).

There were no significant correlations between different executive variables and the values of the ADHD scales in the ASD- and in the TD group.

In contrast to the ADHD symptomatology, we tested the relationship between EF and autistic symptoms (mean impairment of social interaction, mean impairment of communication, mean stereotype behaviour and total score ASD) with the help of Pearson product-moment correlations for the four groups. We found significant correlations in the ASD – group for the variable "inhibition hits" with impairment of social interaction (r = -0.7, p < .000) and ASD total score (r = -0.5, p = .02), for the variable "inhibition omissions" and all ASD subscale scores (impairment of social interaction: r = 0.6, p = .004; impairment of communication: r = 0.4, p = .03; stereotype behaviour: r = 0.5, p = .02; ASD total score: r = 0.5, p = .006) as well as for the variable "flexibility test duration" and "flexibility stages" with stereotype behaviour (r = 0.5, p = .03; r = .51, p = .02).

The ASD+ group showed significant correlations for the variable "flexibility test duration" and "flexibility stages" with stereotype behaviour (r = -0.5, p = .03; r = -0.4, p = .04).

In the ADHD group we found significant correlations for the inhibition paradigm (hits/stereotype behaviour: r = 0.4, p = 0.4, errors/impairment of social interaction: r = -0.4, p = .03; errors/ASD total score: r = -0.5, p = .02; omissions/stereotype behaviour: r = 0.6, p = .007).

There were no significant correlations in the TD group.

## Discussion

The aims of this study were twofold: to investigate profiles of EF (inhibition, flexibility, working memory and planning) in ADHD and ASD with special regard to the comorbidity of ADHD in ASD children and to investigate whether ADHD and ASD symptoms are associated with the applied EF tasks in the four diagnostic groups.

With regard to the first aim, we found clear deficits in inhibition and working memory tasks in the ADHD group, whereas the ASD children showed deficits in flexibility and in planning tasks. ASD+ children were compared to those of the ASD- group particularly impaired in inhibitory performance and flexibility as well as test duration on all the tasks. How do these results fit in with our predictions based on the literature?

### Inhibition task (Go/NoGo-Task)

The expectation that the ADHD children would be more impaired in inhibitory control, especially with regard to errors of omission and commission, was confirmed for the Go/NoGo-task for all variables. Our initial prediction that ASD+ children would show inhibition deficits according to an additivity hypothesis was partly confirmed, as these children showed worse performance making more errors of omission and less hits compared to the healthy control children. Our results are partly in line with findings of previous studies. Ozonoff and Happé found more deficits in response inhibition for ADHD children than for autistic children comparing ADHD and ASD groups, whereas the Nyden, Johnson and partly the Geurts study revealed also deficits for ASD children [12-17]. However less severe inhibition deficits in children with ASD were consistently found in all the above mentioned studies, except of the one by Ozonoff et al. and Goldberg et al. who applied a stroop task [12,15]. Our results confirm a suggestion of Goldberg et al. to better use non-verbal measures (e.g. a Go-NoGo task) to differentiate between ADHD and ASD. A study by Christ et al. assessing children with ASD with different inhibitory tasks revealed that the stroop task didn't lead to inhibition deficits compared to a flanker and a GoNo/Go task [47]. The authors argue that referring to a model by Casey et al., it is possible that the integrity of some but not all neural circuits subserving inhibitory control are compromised in children with ASD [48,49].

An interesting finding of our study is that comorbid ADHD symptoms seem to worsen inhibition performance in ASD children with comorbid ADHD, as pure ASD children performed rather well in comparison to the pure ADHD group in our study. Previous studies including comorbid ADHD symptoms couldn't find differences between ASD and ADHD children and vice versa. This underlines the importance of taking into account severe inattention and hyperactivity problems warranting an ADHD diagnosis in ASD children when interpreting inhibition data. If comorbid ADHD symptoms are not statistically referred to individual inhibition problems of children with ASD might not be detected due to a too large heterogeneity of the samples.

### Intra-Dimensional/Extra-Dimensional Shift Task (ID/ED)

The results for the flexibility task show differences on the basis of effect sizes. The ASD- group made less errors than the control group. As also discussed by Happé one reason for the absence of differences between the ASD and the TD group might be the high proportion of participants with Asperger syndrome [16]. With regard to the variable stages the ASD+ group even performed better than the TD group. To the authors' knowledge there are to date no studies showing that children with Asperger syndrome are better in cognitive flexibility than typically developing children. The ASD+ group, as was the case in the planning task, showed difficulty with test duration compared to the control and the ASD- group. As also the ADHD group had a longer test duration it seems that time is a key problem for those children affected by ADHD-symptoms. Studies using the same task of the CANTAB also failed to find significant differences in post-hoc tests [15,16]. Interestingly, Ozonoff et al. pointed out that not all types of attention-shifting are impaired in ASD, only those that require prefrontal cortical function [12]. An analysis of performance at different cognitive levels of the same flexibility task of the CANTAB revealed no impairment on higher levels when shifting between categories or when rules were required. This kind of analysis was not applied neither in our study nor in the Goldberg or Happé study. Geurts et al. describe for the HFA group slower mean reaction times on a different flexibility measure (change task) and a higher percentage of perseverative responses in the WCST (Wisconsin Card Sorting Test) [14]. Perseveration itself is not measured with the ID/ED Task of the CANTAB.

### Spatial Working Memory Task (SWM)

Even though both groups affected by ADHD needed more time to perform the working memory task, especially in the ADHD-group medium to high effect sizes are apparent with poorer performances in comparison to the control group, whereas pure autistic children also made more errors than the healthy children. Happé et al. also describe deficits on the same working memory task for the ADHD group but not for the ASD group, whereas Goldberg et al. found deficits for both groups with poorer performance of the autistic children [15,16]. One reason why our results are different from the Goldberg study might be that in their study, the ASD children had significantly lower IQs than the ADHD children, whereas we used IQ-corrected z-scores. The result by Geurts et al. who found no differences between an ADHD, ASD and a control group for a different working memory task (self-ordered pointing task) could not be replicated [14].

As we found only small problems of working memory in both ASD groups, comorbid ADHD symptoms don't seem to play the key role in working memory performance. One could argue that there were no effects in the ASD groups due to a wide age range in our study (6–18 years) as age-related improvement for the working memory task was described in particular for ASD children in an analysis of developmental age [16]. This difficulty was eliminated by using age-corrected z-scores in our study.

### Planning Task (Stockings of Cambridge, SOC)

With regard to the performance in the planning task, our results are difficult to interpret. Even effect sizes revealed only medium effects for the ASD+ group concerning duration of the task and medium effects for the mean initial thinking time and the mean subsequent thinking time especially for the ASD- group. ASD+ children also showed deficits on these variables. These results are partly in line with a study by Ozonoff et al., who applied the CANTAB planning task in a large sample of 79 autistic individuals, finding no differences for the mean initial thinking time, but for the mean subsequent thinking time [12]. The number of solved problems was not affected by this and replicates the results of a study by Goldberg et al., who also failed to find group differences in the number of problems solved using the same paradigm [15]. Although the Geurts group used the Tower of London as a different measure of planning abilities they also found significant differences for execution time, with worse performance in a pure autistic group [14]. Thus, planning difficulties for ASD individuals might be less a problem of comprehension than of speed.

### Relationship between EF and ADHD/ASD symptoms

Our second aim was to examine relationships between EF and clinical symptoms of ADHD and ASD. In our study clinically observed ADHD symptoms don't correlate with EF deficits in ADHD children, whereas inhibition performance shows an interaction of comorbid ADHD symptoms in ASD children on all measures, especially with the symptom of inattention. Also test duration seems to be influenced by ADHD symptoms in this group.

Interestingly even though there were low ASD scores in the ADHD sample, inhibitory parameters are associated with ASD symptoms in the ADHD group. Correlations of ASD symptoms and EF don't seem to follow a fixed pattern in the ASD groups.

These results underline the difficulty of bringing together clinically observed behaviour and neuropsychologically measured EF functions. This indicates a need for caution when attempting to transfer laboratory outcomes to daily life.

Due to the small sample size, this investigation can only be seen as a descriptive attempt to approach the problem of influence of attention disorders and increased impulsivity and hyperactivity as postulated for example by Geurts et al. [14]. However the fact that though some aspects of group differences could not be shown by the analysis of variance, it is obvious that there is a high amount of medium and high effect sizes describing to a certain degree differences between the four groups. Therefore it can be hypothesized that the statistical power in our study is too small and that within a larger sample existing differences might be proved as being statistically significant.

Finally the characteristics of our study sample (age- and IQ-correction, inclusion of ADHD in the ASD group) limit the comparability of these findings with respect to other research reports with differently characterized samples.

One reason for our decision to control for IQ, well knowing that there is a current controversy about this topic, was the fact that we partly wanted to avoid issues concerning late maturation of the frontal lobes and the close overlap between the constructs of EF and fluid intelligence [50,51]. Thus, as also argued by Happé et al., findings from studies not controlling for IQ are difficult to interpret [16]. Furthermore especially in ASD samples a number of high EF tasks have shown deficits in low-but not in high-functioning groups with ASD. Finally Hill & Bird point out that the approach of comparing data between single groups is problematic since individuals differences are large and requires that all individuals are homogeneous [3]. Controlling for IQ thus reduces the heterogeneity of the participants.

## Conclusion

This is the first study investigating specifically the impact of comorbid ADHD-symptoms in children with high-functioning ASD on EF performance. To the authors knowledge this is the first study using a four-sample design including two ASD groups (with and without comorbid ADHD symptoms). Our hypothesis that ADHD children are more impaired in inhibition and working memory tasks whereas ASD children show more deficits in planning and flexibility abilities were confirmed. The hypothesis concerning flexibility was partly confirmed as only ASD children with comorbid ADHD had deficits in this task.

The additivity hypothesis (see Introduction section) saying that the ASD+ group performs worse by an order equivalent to the addition of each disorder individually was partly confirmed with clear deficits in inhibitory performance of these children compared to the ASD- group. However this effect could not be found for the working memory task. The hypothesis was confirmed as in planning and flexibility abilities there were no additional deficits than those presented by the autistic children themselves.

Furthermore the study showed that individuals with ASD and comorbid ADHD have more of a speed than a comprehension problem in planning, working memory or flexibility tasks.

In conclusion the paper shows that ASD related studies should take symptoms of ADHD into account as the dimensional overlap in EF functions in ADHD and ASD with comorbid inattention and hyperactivity symptoms might be used to describe an endophenotype. As also stated by Verté et al. "dimensional (or even multivariate) approaches should be employed in order to understand the relationship between multiple overlapping endophenotypes in more depth" [52]. Genetic and environmental risk factors and the pathophysiological mechanisms of ADHD and ASD should be specified to "target a more homogenous piece of the etiological puzzle of these disorders", as suggested for ADHD by Doyle et al. [53].

## Competing interests

The author(s) declare that they have no competing interests.

## Authors' contributions

JS participated in the design and coordination of the study, performed the statistical analysis and wrote the manuscript.

DM and NB carried out the recruiting and neuropsychological assessment of the participants and contributed to the interpretation of the data.

MS revised the manuscript.

GL has been involved in designing the study and revising the manuscript. He gave final approval of the final version to be published.

All authors read and approved the final the final manuscript
